# Editorial: Prostate Cancer: What We Know and What We Would Like to Know

**DOI:** 10.3389/fonc.2015.00114

**Published:** 2015-05-22

**Authors:** Gianluigi Taverna, Richard J. Cote, Fabio Grizzi

**Affiliations:** ^1^Department of Urology, Humanitas Clinical and Research Center, Milan, Italy; ^2^Department of Pathology, Dr. John T. Macdonald Foundation Biomedical Nanotechnology Institute, University of Miami Miller School of Medicine, Miami, FL, USA; ^3^Department of Inflammation and Immunology, Humanitas Clinical and Research Center, Milan, Italy

**Keywords:** prostate, cancer, diagnosis, biomarkers, angiogenesis, radiotherapy

Cancer research has undergone radical changes over the last few years. The issue today is no longer the amount of molecular, cellular, and clinical information available, but how to handle it. Systems biology is the latest in a series of innovative strategies driven by technological advances that have provided us with a suite of “omics” (1). Technology advancements have enabled comprehensive characterization of genomic, epigenomic, transcriptomic, proteomic, and metabolomic alterations in tumor specimens (2). Despite this continuous progress, prostate cancer remains a major public health problem throughout the world (3). Among men, cancers of the prostate, lung and bronchus, and colon–rectum will account for about 50% of all newly diagnosed cancer (4). Prostate-specific antigen (PSA) is one of the most widely used tumor markers. Although its possible use in early diagnosis or screening PSA has various drawbacks mainly due to its low specificity (5) considering that more than half of the men with PSA over 4.0 ng/ml are negative on initial biopsy (6). A new generation of biomarkers is emerging, consisting of genomic-, serum-, urine-, and tissue-based assays that may supplement PSA testing, or replace it over time (7–9). Although the identification and development of more specific diagnostic biomarkers are considered important, the major focus in translational prostate cancer research is the identification of potential “quantitative variables” to detect clinically significant prostate cancers. As reported by Vlaeminck-Giullem et al. (10), the current dilemma is to specifically distinguish among all prostate tumors the very aggressive high-grade cancers that will become life threatening by developing extra-prostatic invasion and metastatic potential from the indolent cancers that will never modify a patient’s life expectancy. At molecular level, Src and other members of the Src kinase family have been proposed as potential candidates (10). Recently, it has been shown that the activation of Src-dependent intracellular pathways is frequently observed in clinically significant prostate cancer (11). The proto-oncogene tyrosine-protein kinase Src influences some of most important events that accompany tumor progression, including cell proliferation, cell motility, invasion, epithelial-to-mesenchymal transition, resistance to apoptosis, angiogenesis, neuroendocrine differentiation, and metastatic diffusion (11). The particularity of the Src oncogenic action in prostate carcinogenesis is its ability to interfere with the androgen pathway. Through both direct and indirect interaction with the androgen receptor (AR), Src reinforce the proliferative and anti-apoptotic actions of the AR, even in the absence of specific ligands. These molecular mechanisms constitute a solid rationale in favor of the use of Src inhibitors in managing patients with prostate cancer (10, 12). On the other hands, decades of research are now leading to therapeutics that target the molecular mechanisms of the cancer-specific immune response (13). These therapeutics include tumor antigen vaccines, dendritic cell activators, adjuvants that activate innate immunity, adoptive cellular therapy, and checkpoint blockade (13). Another major effort has been in prostate cancer prevention, where blockade of the testosterone metabolism pathway may be a major focus. However, the results of two large randomized, placebo-controlled trials: the prostate cancer prevention trial (PCPT) with finasteride and the reduction by dutasteride of prostate cancer events (REDUCE) trial have fueled controversies on the use of 5α-reductase inhibitors (5-ARI). Finasteride and dutasteride are among the agents used to target the androgen–AR axis to prevent the development and/or progression of prostate cancer, as summarized by Kosaka et al. (14). In particular, 5-ARIs have been shown to play a potential role in preventing clinical progression among men with low-risk prostate cancer on active surveillance. However, in light of the US Food and Drug Administration recommendation against 5-ARIs for primary chemoprevention, these findings should be interpreted with caution (3, 15).

It is recognized that prostate cancer can mainly be diagnosed on the basis of increased PSA levels associated with a low accuracy of the randomly sampled biopsy fragments and the well-known subjectivity of a pathologist’s interpretation (16). New imaging strategies include multiparametric magnetic resonance imaging (MRI) and MRI/ultrasound (US) fusion. Multiparametric MRI combines T2-weighted imaging, diffusion weighted imaging, and perfusion imaging. Published data suggest that multiparametric MRI guided biopsy results in the increased detection of more clinically significant high-grade cancers, with lower detection of low-grade clinically indolent cancers (16, 17). However, a high prevalence of both overuse and underuse of guideline-recommended imaging in prostate cancer has been observed, and additional research is required to examine contributing factors to guideline non-adherence in the imaging work-up of prostate cancer (18). As reported by Giannarini et al. (19), multiparametric MRI should be further evaluated in different clinical scenarios in which it is desirable to reduce the proportion of unnecessary prostate biopsies and to limit the detection of indolent disease, such as opportunistic screening, persistent prostate cancer suspicion in men with previous negative prostate biopsies, and eligibility for active surveillance. Continued improvement in standardization of technical parameters, functional sequences, and image reporting systems is a pre-requisite for a rapid and successful dissemination of this imaging modality (19). Recently, Hoeks et al. performed in-bore MRI guided biopsies in 265 patients with elevated PSA and previous negative TRUS-guided biopsies (20). Prostate cancer was found in 41% of patients and the majority of the detected cancers (87%) were clinically significant (20). However, in-bore MRI guided biopsies have some limitations, including discomfort related to patient position, increased costs related to long procedure duration, and the requirement for special non-magnetic equipment (17). Furthermore, the actual classification of very low-, low-, intermediate-, and high-risk disease is only accurate in the case of patients with high-risk disease. Other factors such as early detection, population aging, and better treatment have contributed to increasing the prevalence of prostate cancer, thus fueling a need for the continuous monitoring of prevalence indicators in order to identify needs, plan the allocation of resources, and improve healthcare programs for cancer survivors.

Angiogenesis, the development of new branching vessels from existing vasculature, is a complex process observed in fetal growth, wound healing, endometrial hyperplasia associated with the menstrual cycle, and in many diseases, including cancer (21). The significance of angiogenesis in prostate cancer still remains controversial (22–24). Štifter and Dordević (25) try to express opinion that in near future we should search for the next generation of promising therapeutics for the management of prostate cancer. Till now, several agents have been approved by the food and drugs administration (FDA) but their efficacy is still limited and this observation is repeatedly proven by recurrence of more aggressive tumor after application of anti-angiogenic therapy (25). Alternatively, they proposed two options of therapeutic actions, one targeting reactive stroma and other is aiming at inflammatory, stromal, and circulating cells (25).

The Research Topic entitled “Prostate Cancer: what we know and what we would like to know” has highlighted some molecular, cellular, clinical, epidemiological, and imaging findings that are fundamental for identifying the patients who are in most need of aggressive therapeutic intervention, viewing prostate cancer as a system that is dynamically complex. It is now widely accepted that prostate cancer encompasses various pathological entities and a wide range of clinical behaviors, and is underpinned by a complex array of genetic alterations that affect supra-molecular processes (26, 27). It is this genetic and phenotypical variability that primarily determines the progression of prostate cancer and its response to therapy. Furthermore, the asynchrony and self-progression of prostate cancer cell populations suggests that the extent to which each neoplastic cell shares the properties of a natural cell differs in terms of time and space. Individual cells from a clonal cell population may respond differently (or not at all) to the same stimulus. Prostate cancer consists of distinct subpopulations of cancer cells, each with its own characteristic sensitivity to a given therapeutic agent. Cancer therapies can therefore be seen as filters that remove the sensitive subpopulations, but allow insensitive subpopulations to escape (28). Noteworthy, in 2011, Cornù et al. have shown that dogs can be trained to detect prostate cancer by smelling urine with a significant success rate (29). By analyzing 33 patients with prostate cancer, they reported a sensitivity and specificity of 91%, and suggested that prostate cancer gives an odor signature to urine (29). Recently, Taverna et al. (30) established that a trained canine olfactory system can detect prostate cancer-specific volatile organic compounds (VOCs) in urine samples. Their study included 902 subjects (362 with prostate cancer ranging from very low risk to metastatic and 540 healthy, affected by non-neoplastic diseases or non-prostate cancer control participants). The urine samples from both groups were blinded and analyzed by two dogs, and the sensitivity, specificity of each dog’s efficiency was assessed. The first dog achieved a sensitivity of 100% and a specificity of 98%. The second dog reached 98.6% specificity, 97.6% sensitivity (30). These data encourage all of us to redouble our efforts to improve the detection of prostate cancer (31). Unanswered questions, however, remains, including what does the dogs smell? Further studies should be designed to research whether a single odor or a mixture of prostate cancer-specific VOCs are recognized by the dogs. Another question is: “How could a dog that detects prostate cancer-specific VOCs be used in daily practice?” The potential predictive power of this method needs to be additionally investigated by studying patients with negative biopsies, elevated PSA serum value, and an adequate follow-up. It has been stated that the identification of the VOCs could lead to a potentially useful screening tool for prostate cancer (29). Additionally, this approach might have the potential to offer a non-invasive alternative to PSA sampling and prostate biopsy for detecting prostate cancer (32). It is known that biomarkers are features that can be objectively measured and evaluated as indicators of normal processes, pathogenic processes, or pharmacologic responses to a therapeutic intervention, and that biomarkers can be clinical parameters, laboratory measures, imaging-based measures, or genetic and molecular determinants. It is indubitable that the combined efforts of urologists, oncologists, pathologists, biologists, radiotherapists, and mathematicians can contribute much toward improving our understanding of the complexity of cancer, and such a multidisciplinary approach will help to clarify existing concepts, categorizes current knowledge, and suggest alternative approaches to the discovery of biomarkers and predictive values that urgently need to be translated into clinical practice.

## Conflict of Interest Statement

The authors declare that the research was conducted in the absence of any commercial or financial relationships that could be construed as a potential conflict of interest.
